# Accumulation of Glycogen and Upregulation of LEA-1 in *C. elegans daf-2(e1370)* Support Stress Resistance, Not Longevity

**DOI:** 10.3390/cells11020245

**Published:** 2022-01-12

**Authors:** Aleksandra Zečić, Ineke Dhondt, Bart P. Braeckman

**Affiliations:** Laboratory of Aging Physiology and Molecular Evolution, Department of Biology, Ghent University, K. L. Ledeganckstraat 35, B-9000 Ghent, Belgium; aleksandra.zecic@uk-koeln.de (A.Z.); ineke.dhondt@anacura.com (I.D.)

**Keywords:** *C. elegans*, insulin/IGF-1, *daf-2(e1370)*, longevity, stress resistance, glycogen, LEA-1

## Abstract

DAF-16-dependent activation of a dauer-associated genetic program in the *C. elegans* insulin/IGF-1 *daf-2(e1370)* mutant leads to accumulation of large amounts of glycogen with concomitant upregulation of glycogen synthase, GSY-1. Glycogen is a major storage sugar in *C. elegans* that can be used as a short-term energy source for survival, and possibly as a reservoir for synthesis of a chemical chaperone trehalose. Its role in mitigating anoxia, osmotic and oxidative stress has been demonstrated previously. Furthermore, *daf-2* mutants show increased abundance of the group 3 late embryogenesis abundant protein LEA-1, which has been found to act in synergy with trehalose to exert its protective role against desiccation and heat stress in vitro, and to be essential for desiccation tolerance in *C. elegans* dauer larvae. Here we demonstrate that accumulated glycogen is not required for *daf-2* longevity, but specifically protects against hyperosmotic stress, and serves as an important energy source during starvation. Similarly, *lea-1* does not act to support *daf-2* longevity. Instead, it contributes to increased resistance of *daf-2* mutants to heat, osmotic, and UV stress. In summary, our experimental results suggest that longevity and stress resistance can be uncoupled in IIS longevity mutants.

## 1. Introduction

In the long-lived *Caenorhabditis elegans daf-2* mutant, activation of transcription factor FoxO/DAF-16 elicits a genetic program that leads to vast dauer-like changes in the worms’ physiology [1,2,3,4,5]. These include a strong decrease in protein turnover rates and increased proteostasis [6,7,8,9,10], enhanced stress resistance and innate immunity [11,12,13,14], and extensive restructuring of intermediary metabolism [15,16,17,18,19]. One of the major changes in *daf-2* metabolism is accumulation of excessive amounts of glycogen in the intestine, hypodermis and body wall muscles [5,20], in accord with elevated levels of glycogen synthase (GSY-1) [5]. Similarly, ultrastructural studies in dauer larvae reported the presence of glycogen deposits in the pharynx and body wall muscles, as well as neurons [21]. In the non-feeding dauers of *C. elegans*, as well as the two entomopathogenic nematodes, *Steinernema carpocapsae* and *S. feltiae*, glycogen serves as an easily mobilized source of energy that fuels locomotion and nictation behaviour, and contributes to nematode infectivity, respectively [21,22]. Furthermore, glycogen was shown to mitigate hyperosmotic stress in wild-type *C. elegans* by acting as a readily available source for rapid synthesis of glycerol [23]. Stress-protective roles of glycogen were also demonstrated in wild-type worms exposed to oxidative stress [24], anoxia [20] and combined hypoosmotic-anoxic stress [25]. The latter stress was also detrimental for *daf-2* mutants with depleted glycogen reserves due to *gsy-1* knockdown [25]. Thus, it appears that glycogen could play a major role in enhanced resistance to different environmental insults in *daf-2* worms. On the other hand, its role in *daf-2* longevity is less clear. It is conceivable that glycogen could function as a short-term energy source to support longevity, similarly as in dauers. However, one study showed a negligible reduction in *daf-2* lifespan due to *gsy-1* knockdown [24], whereas the other even reported a lifespan-extending effect of the same treatment [26].

Two independent proteomic studies in *daf-2* mutants have also identified increased abundance of a late embryogenesis abundant protein LEA-1 [5,16], which belongs to the group of intrinsically disordered proteins found in bacteria [27], algae [28], plants [29,30], nematodes [31,32,33], and some crustaceans, such as *Artemia franciscana* [34,35]. LEA proteins play an important role in protecting cells against several abiotic stresses, such as desiccation, high salinity, heat and cold stress [36,37]. Furthermore, it has been shown that LEA proteins act in concert with trehalose to prevent desiccation- and heat-induced protein aggregation in vitro [38]. In *C. elegans* dauer larvae, LEA-1 is essential for desiccation tolerance, and survival of heat and osmotic stress [33,39]. The relevance of this protein for *daf-2* longevity and stress resistance was not previously assessed, however, it is plausible that upregulation of *lea-1* could play a similar role as in dauers, at least in part through synergistic action with trehalose.

Considering our most recent findings that trehalose accumulation is dispensable for *daf-2* longevity but contributes to its increased tolerance to hyperosmotic stress and, to a limited extent, heat stress [40], we asked whether accumulation of glycogen and upregulation of *lea-1* similarly have a sole purpose of protection against stress, that is unrelated to lifespan extension in *daf-2* mutants.

## 2. Materials and Methods

### 2.1. C. elegans Strains and Culture Conditions

*C. elegans* strains were maintained at 16 °C on nutrient agar (Oxoid Ltd. Thermo Fisher Scientific Inc., Basingstoke, Hampshire, UK) plates seeded with *E. coli* OP50 as a food source. The worm strains used in this study are Bristol N2 wild-type and CB1370 {*daf-2(e1370)* III}.

Both for culturing and experimental purposes, worms were synchronized at the first larval stage (L1) by treating gravid hermaphrodites with alkaline hypochlorite solution [41]. To allow hatching, the obtained eggs were incubated overnight in S buffer (0.1 M NaCl and 0.05 M potassium phosphate buffer pH 6.0) at 20 °C with shaking (100 rpm).

### 2.2. RNAi Treatment

To knock down genes of interest, RNAi by feeding was performed, with slight modifications of a previously described protocol [42]. *E.coli* strains (HT115) expressing dsRNA against target genes were obtained from the Ahringer [43] and Vidal RNAi feeding library [44] (distributed by Source BioScience). The strain carrying the empty vector L4440 was used as a control. RNAi bacteria were grown overnight at 37 °C in LB medium containing 50 μg/mL carbenicillin and 12.5 μg/mL tetracycline. These bacteria were then seeded onto nematode growth medium (NGM) agar plates supplemented with 25 μg/mL carbenicillin and 1 mM isopropyl-β-d-thiogalactopyranoside (IPTG), and the induction was performed overnight at room temperature. NGM plates were allowed to dry for 3–5 days at room temperature before seeding. All RNAi clones were verified by DNA sequencing.

### 2.3. Lifespan Assay

After hatching in S buffer, L1 larvae were transferred to NGM plates seeded with 100 µL of corresponding RNAi bacteria and grown at 16 °C to L4 larval stage in order to avoid dauer formation in *daf-2(e1370)* mutants. At L4 larval stage, worms were shifted to 20 °C for the remainder of the experiment. RNAi knockdown was initiated from L1 stage. The lifespan measurements were initiated at day 0, which was defined as the day of L4-to-adult transition. During the reproductive period, worms were transferred daily to fresh plates by picking, and from then on, every other day until day 12 and day 20 (wild-type and *daf-2(e1370)* background, respectively). Survival was scored every other day, and the worms were considered dead when not responding to gentle prodding with a platinum wire. Worms that crawled off the plate or died due to protruding vulva or bagging were censored. The experiments were repeated at least 2 times independently, and for each condition ~120 worms were followed. Survival curves were generated using GraphPad Prism 9 (version 9.2.0, GraphPad Software Inc., San Diego, CA, USA). For statistical analysis, a Mantel–Cox test was performed using the online application for survival analysis 2 (OASIS2) [45].

### 2.4. Stress Resistance Assays

For all stress resistance assays, worms were grown at 16 °C on RNAi plates until L4 stage, at which point they were treated with 100 µM FUdR and transferred to 20 °C until day 2 of adulthood.

#### 2.4.1. Heat and Oxidative Stress Assays

Resistance to heat and oxidative stress was assessed by the label-free automated survival scoring (LFASS) approach, which relies on time-lapse measurements of blue (death) fluorescence (λ_ex_ = 360 nm, λ_em_ = 435 nm) that is generated at the onset of the worms’ death [46]. The assays were run in black clear-bottom 96-well plates (Greiner Bio-One International GmbH, Kremsmünster, Austria) which were placed in a Tecan Infinite NANO+ plate reader (Tecan Group Ltd., Männedorf, Switzerland). For both assays, 100 µL of worm suspension in S buffer (~200 worms) was transferred into each well, together with previously frozen HT115 *E. coli* (L4440) to avoid starvation. The plates were covered with a lid and sealed with parafilm. To prevent the plates from drying out in the heat shock experiments, empty wells were filled with water. Blue fluorescence was measured for each well in two-minute intervals over the span of 24 h. To examine their resistance to heat and oxidative stress, the worms were exposed to 40 °C and 0.28% *tert*-butyl hydroperoxide (TBHP; Merck KGaA, Darmstadt, Germany), respectively. Median time of death, which corresponds to half-maximal blue fluorescence, was automatically extracted for each well in MATLAB 9.9 (The MathWorks, Inc., Natick, MA, USA), using the LFASS software package developed by Benedetto et al. [46]. Both stress assays were run in three independent trials, each with 3–5 technical replicates (wells) per experimental condition. GraphPad Prism 9 (GraphPad Software Inc., USA) was used for statistical analyses and to generate graphs. Statistical significance was assessed by two-way ANOVA.

#### 2.4.2. Osmotic Stress Assay

To assess the resistance to chronic hyperosmotic stress, day 2 adults were manually transferred onto NGM plates containing 500 mM NaCl [47] and 100 µM FUdR. Survival was scored daily until all the worms with wild-type background died, and thereafter, every 2–3 days until the end of the experiment. Each experiment was repeated 2–3 times independently, with approximately 120 animals per trial for each condition (~20 worms per plate). Survival curves were generated using GraphPad Prism 9 (GraphPad Software Inc., USA). For statistical analysis, Mantel–Cox test was performed using the online application for survival analysis 2 (OASIS2) [45].

#### 2.4.3. UV Stress Assay

For UV treatment, we followed a previously described protocol [48], with slight modifications. Namely, day 2 adults were washed twice in S buffer to remove bacteria and placed onto fresh NGM plates without food so as to avoid blocking of UV rays. The animals were irradiated with germicidal UV light (254 nm) in a Stratalinker 2400 (Stratagene, La Jolla, CA, USA) at a dose of 200 J/m^2^. After 1 h recovery in the dark, ~120 worms per condition were manually transferred onto RNAi plates containing 100 µM FUdR, and their survival was followed daily. The experiments were repeated at least 2 times independently, with 6 plates per condition per experiment (~20 worms per plate). For statistical analysis, Mantel–Cox test was performed using the online application for survival analysis 2 (OASIS2) [45], and the survival curves were generated using GraphPad Prism 9 (GraphPad Software Inc., USA).

### 2.5. Determination of Trehalose and Glycogen Content

Approximately 2000 L1 larvae were placed onto each NGM plate seeded with 500 µL of 5× concentrated RNAi bacteria and grown at 16 °C until the L4 stage. L4 worms were washed off onto fresh RNAi plates containing 100 µM FUdR and shifted to 20 °C until day 2 of adulthood. Day 2 adults were washed twice in S buffer and once in 2.5 mM EDTA in S buffer to remove bacteria. After 2 more rinses with double distilled water (ddH_2_O), 100 µL of a dense worm pellet was collected in each sampling tube, flash frozen in liquid nitrogen and stored at −80 °C until further use. Each experimental condition was sampled in 3 biological replicates.

For analysis, 150 µL of ddH_2_O was added to frozen samples, together with approximately 200 mg of glass beads (~0.25 mm), and homogenisation was performed by bead-beating at 6800 rpm for 30 s in a Precellys 24 tissue homogenizer (Bertin Technologies, Montigny-le-Bretonneux, France). Debris was pelleted by centrifugation at 14,000 rpm (20,800× *g*) for 5 min at 2 °C, and the obtained supernatant was used for subsequent analyses. Then, 10 µL of the supernatant was used to quantify the total protein content in the sample with a Bicinchoninic acid (BCA) Assay Kit (Thermo Fisher Scientific Inc., USA), following the manufacturer’s instructions. The rest of the supernatant was heated to 95 °C for 10 min in a heat block, and thereafter 60 µL and 10 µL of the sample were used for trehalose and glycogen assays, respectively. Thus, both sugars were quantified from the same initial worm sample, and the assays were run simultaneously.

Trehalose levels were quantified in a 96-well plate (Greiner Bio-One International GmbH, Austria) using the Trehalose Assay Kit (Megazyme Ltd., Wicklow, Ireland) as described previously [40].

For determination of glycogen content, the Glycogen Assay Kit (Sigma-Aldrich, St. Louis, MO, USA) was used according to the manufacturer’s instructions. Briefly, samples were prepared as described above, diluted in ratio 1:10, and 10 µL of diluted sample was added per well of the 96-well plate (Greiner Bio-One International GmbH, Austria). After 30 min incubation with hydrolysis enzyme mix, 50 µL of master reaction mix (containing the fluorescent peroxidase substrate) was added to each well. The absorbance was measured at 570 nm with Tecan Infinite M200 Plate Reader (Tecan Group Ltd., Switzerland) at 24 °C, and in 1 min intervals over the span of 90 min. The absorbance value from the plateau phase of the reaction was used to determine the quantity of glycogen in each sample from a standard curve. Each sample was run in 2 technical replicates (wells), and each assay was run 3 times independently. Glycogen levels were normalized to the total protein content in the sample.

For both assays, GraphPad Prism 9 (GraphPad Software Inc., USA) was used to perform statistical analyses and produce graphs. Statistical significance was assessed by two-way ANOVA.

### 2.6. Oil Red O (ORO) Staining and Quantification

Approximately 4000 worms per experimental condition were grown similarly as described above. Half of the animals were grown until day 2 of adulthood on RNAi plates seeded with corresponding bacteria, while the rest were transferred to culture flasks as day 1 adults and starved for 44 h in S buffer supplemented with 100 µL FUdR. The amount of neutral lipids was determined by ORO staining, which was performed according to the protocol described in Escorcia et al. [49]. Briefly, animals were washed 3 times in 1× phosphate-buffered saline +0.01% Triton X-100 (PBST) solution and fixed in 40% isopropanol for 15 min. Isopropanol was removed and samples were stained for 2 h in ORO working solution (prepared and filtered the day before by diluting the stock solution in water (3:2) to 60% isopropanol). In the following step, the worms were incubated for 30 min in PBST to remove the excess stain. This step, as well as fixation and staining, were carried out on a rotator. After three additional washes in PBST, about 100 µL of worm suspension was spread on a microscope slide, while minimising the overlap between the animals. Images were obtained using a Nikon SMZ-745T stereomicroscope (Nikon Instruments Europe BV, Amsterdam, The Netherlands) with a ToupTek XCAM 1080 PHA digital camera (ToupTek Photonics, Hangzhou, Zhejiang, China) at 20× magnification. The experiments were performed three times independently. Per each biological replicate, at least 20 images were taken for each experimental condition, and 5–8 worms were analysed per image.

Image analysis was performed using CellProfiler 4.07 [50] and the fully automated pipeline developed by Wählby et al. [51] with some modifications. In brief, the input colour images were first inverted and then split into red, green, and blue channel components. The green channel was used for ORO quantification. Thereafter, the software identified clusters of worms, and at this step we excluded all the worms that were touching the border of the image, thus ensuring that only complete worms were used for the subsequent quantification. The pre-constructed “untangling” model was then applied to define single worms. Here, we built our own model by manually selecting single non-touching worms that were representative of the variation in worm shape and posture within our dataset. As the final step in the pipeline, the software extracted the mean intensity of ORO staining on a per-worm basis. For each tested condition, the data were collected from hundreds of worms across 3 independent experiments. The individual measurements were then imported and sorted in Microsoft Excel (version 2111 (build 16.0.14701.20254), Microsoft Corporation, Redmond, WA, USA) and GraphPad Prism 9 (GraphPad Software Inc., USA) was used for subsequent statistical analyses and generation of graphs. The data were not normally distributed, thus, a Kruskal–Wallis test was performed to assess statistical significance, followed by Dunn’s post-hoc test for multiple comparisons.

### 2.7. RNAi Efficiency Quantification by Real-Time Quantitative PCR (RT-qPCR)

The worm samples were obtained as described above. The total RNA was extracted using the column-based RNeasy Mini Kit (Qiagen Benelux BV, Antwerp, Belgium) according to the manufacturer’s instructions, with addition of the DNase I incubation step to remove the genomic DNA contamination. For the DNase I treatment, we used the RNase-free DNase set (Qiagen Benelux BV, Antwerp, Belgium) and followed the manufacturer’s protocol. RNA concentration and purity were determined using a NanoDrop ND-1000 spectrophotometer (NanoDrop products, Wilmington, DE, USA). First strand cDNA was synthesized from 1 μg of RNA using an oligo(dT)18 primer and RevertAid H Minus Reverse Transcriptase (Thermo Fisher Scientific Inc., USA) for 1 h at 42 °C, and the reaction was terminated by heating at 70 °C for 5 min. For each sample, cDNA was synthetised in two separate reactions, which were then pooled prior to RT-qPCR to reduce variation. Primers for *pygl-1* (forward: CGAGAACATCTTCATCTTCGGAATG, reverse: ATCTGGATCTTCTGGAGTGAACATG) and *lea-1* (forward: GAAACAAGATCTCCAATGCGTTCA, reverse: ATCAGACATCTCCTCTCCTGTTTG) were designed with the Primer3 software [52], and manufactured by Invitrogen (Waltham, MA, USA). The primer sequences for *gsy-1* were previously published in [26]. RT-qPCR was run in a Rotor-Gene 2000 centrifugal real-time cycler (Corbett Research, Mortlake, Australia) and the platinum SYBR Green RT-qPCR SuperMix-UDG (Invitrogen, Waltham, MA, USA) was used for detection of the amplification product, as described previously [53]. In each run, we also included a no-template control for every primer pair. To determine the product specificity in each reaction tube, melting curve analysis was performed. The threshold cycle (Ct) values obtained from the Rotor-Gene software version 6 (Corbett Research Mortlake, Mortlake NSW, Australia) were exported to qBase+ software (version 2.3, Biogazelle, Zwijnaarde, Belgium) for further analysis, as described in [53,54]. We used genes *eif-3*, *pmp-3*, *cdc-42* and *iscu-1*, as reference targets for normalisation [53]. The RNAi efficiency was assayed in a single biological replicate, with two technical replicates for each condition.

## 3. Results

### 3.1. Accumulation of Glycogen Plays No Role in daf-2(e1370) Longevity

**Figure 1:**
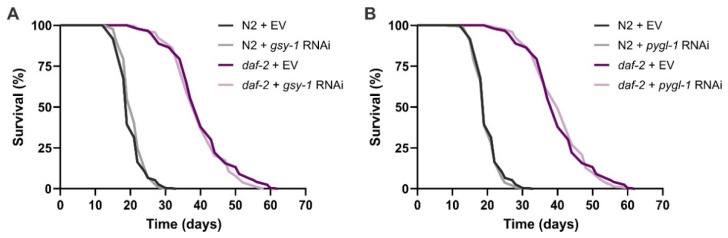
The role of glycogen accumulation in *daf-2(e1370)* longevity: survival curves of wild-type N2 and *daf-2(e1370)* mutants exposed to (**A**) *gsy-1* and (**B**) *pygl-1* RNAi starting from hatching. The graphs represent pooled data from two independent experiments. EV—empty vector control. The data are summarized in Table S1.

In *C. elegans*, like in other animals, excess glucose is stored as glycogen. The rate-limiting enzyme in glycogen synthesis is glycogen synthase (GSY-1) that catalyses the transfer of glucose monomers from uridine diphosphate (UDP)-glucose to the terminal branch of the growing glycogen chain to form α(1→4) glycosidic bonds. Glycogen phosphorylase (PYGL-1), on the other hand, is the key enzyme for glycogen breakdown, which catalyses sequential phosphorolysis of glycogen to release the terminal glucose residue in the form of glucose-1-phosphate [55]. Both GSY-1 and PYGL-1 show increased protein abundance in *daf-2* worms [5], in agreement with large glycogen deposits in the worms’ intestine, hypodermis and muscles. To address the importance of glycogen for *daf-2* longevity, we performed RNAi knockdown of *gsy-1* and *pygl-1* starting from hatching. As our data demonstrate, disruption of glycogen synthesis or breakdown did not affect *daf-2* survival, indicating that this sugar plays no role in *daf-2* lifespan extension (Figure 1, Table S1).

### 3.2. Gsy-1 and Pygl-1 Knockdowns Affect Glycogen, but Have No Impact on Trehalose Levels in daf-2(e1370) Mutants

**Figure 2:**
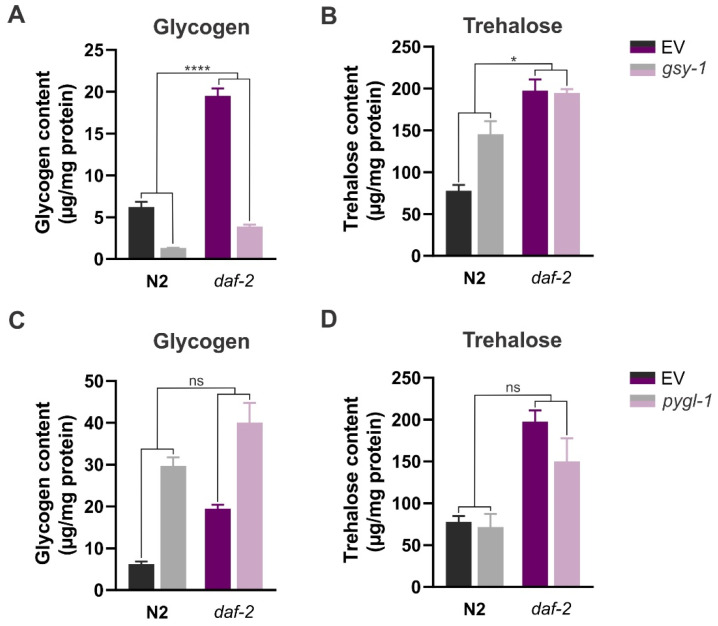
Suppressing glycogen synthesis and breakdown influences carbohydrate levels: glycogen and trehalose content in wild-type and *daf-2(e1370)* mutant treated with *gsy-1* (**A**,**B**) and *pygl-1* (**C**,**D**) RNAi, respectively. Error bars indicate SEM of three independent replicates. EV—empty vector control. ns: *p* > 0.05, * *p* ≤ 0.05, **** *p* ≤ 0.0001 (interaction, two-way ANOVA). The data are summarized in Table S2.

Considering that suppression of *gsy-1* and *pygl-1* did not influence longevity of *daf-2* mutants, we sought to explore the efficiency of the RNAi treatment by quantifying glycogen levels in these worms using standard biochemical assays, and performing the qRT-PCR (Figure S1). We found that glycogen levels in *daf-2* mutants were increased 3-fold in comparison to the wild-type worms (*p*_strain_ < 0.0001; Figure 2A,C; Table S2), which is a stronger phenotype than previously reported by Gusarov et al. [24] and Seo et al. [26]. RNAi knockdown of *gsy-1* resulted in a strong reduction of glycogen content in both strains, i.e., 78% and 80% in N2 and *daf-2*, respectively (*p*_RNAi_ < 0.0001, Figure 2A). As expected, *pygl-1* RNAi had the opposite effect: it caused a further 2-fold increase in glycogen storage in *daf-2* mutants (*p*_RNAi_ = 0.0015), whereas in the wild-type, glycogen content was increased 4.8-fold (*p*_RNAi_ = 0.0003; Figure 2C). Taken together, our data show that *daf-2* mutants do not rely on the total glycogen content to support their longevity.

Furthermore, we explored whether the lack of glycogen or the inability to catabolize it would affect trehalose levels in the worms. We reasoned that glycogen could function as a rapid, proximate source of glucose units for trehalose synthesis. We found 2.5-fold higher trehalose content in *daf-2* mutants in comparison to the wild-type N2 (*p*_strain_ = 0.0004; Figure 2B,D; Table S2), which corroborates previous findings on elevated levels of this disaccharide in *daf-2* worms [19,26,56]. *gsy-1* knockdown did not affect trehalose levels in *daf-2* mutants, while it caused a 1.9-fold increase in the wild-type (*p*_RNAi_ = 0.0156; Figure 2B). These observations agree with the study of Seo et al. [26] that also reported a compensatory switch to storing excess glucose in the form of trehalose when synthesis of glycogen was supressed in the wild-type N2. Moreover, it was shown earlier that supplementing *daf-2* mutants with 5 mM trehalose did not cause a further increase in trehalose stores in these worms [10]. Altogether, these findings suggest that, unlike the wild-type worms, *daf-2* mutants reached the maximum capacity for endogenous trehalose production and storage under normal physiological conditions, that cannot be further enhanced by dietary supplementation or limiting glycogen synthesis. Knocking down *pygl-1* caused a small decrease in trehalose content in *daf-2* mutants (to assess statistical significance of this pairwise comparison with certainty more replicates are needed), with no effect in the wild-type (Figure 2D). However, overall, changes in trehalose levels due to *pygl-1* knockdown were not statistically different in N2 and *daf-2* backgrounds (*ns*, interaction, two-way ANOVA). This suggests that under normal physiological conditions worms do not rely on breakdown of glycogen to support trehalose synthesis.

### 3.3. Suppression of Glycogen Synthesis and/or Degradation Reduces Resistance to Osmotic and Heat Stress in daf-2(e1370) Mutants

**Figure 3:**
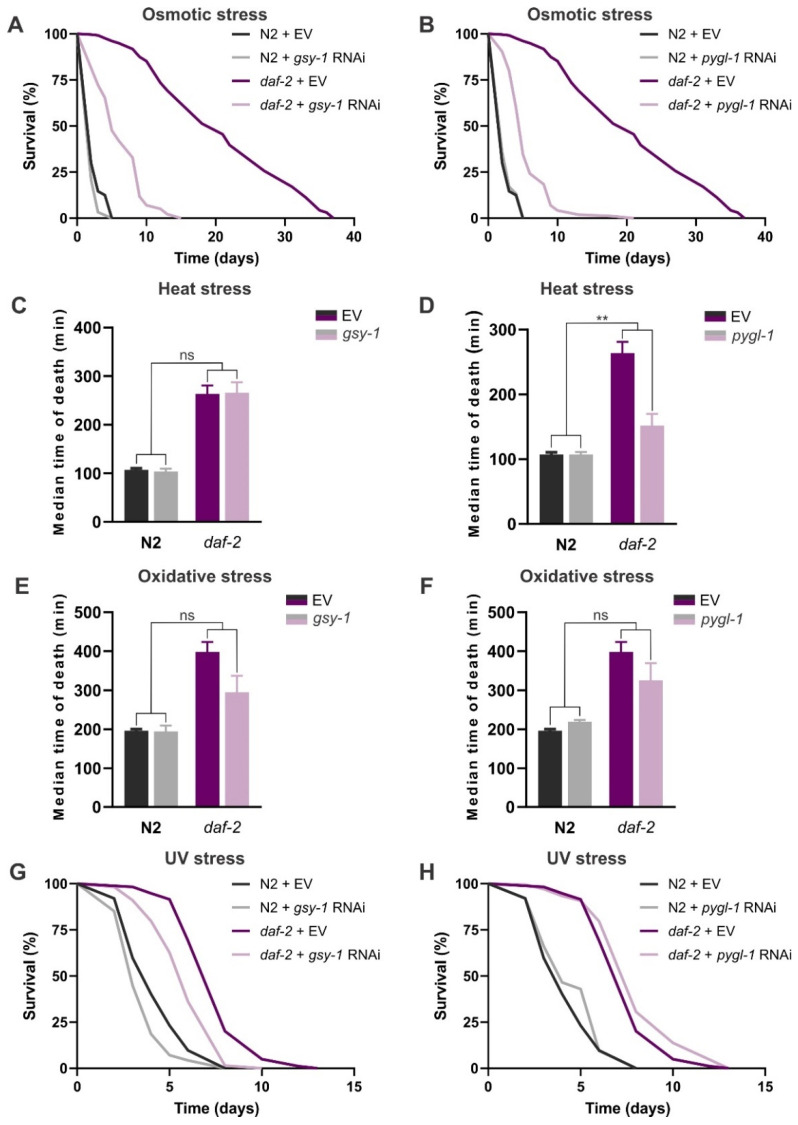
The role of *gsy-1* and *pygl-1* knockdown in stress resistance of wild-type N2 and *daf-2(e1370)*: (**A**,**B**) osmotic stress, 500 mM NaCl; (**C**,**D**) heat stress, 40 °C; (**E**,**F**) oxidative stress, 0.28% tert-butyl hydroperoxide (TBHP); and (**G**,**H**) UV stress (254 nm, 200 J/m^2^). Survival curves in (**A**,**B**) represent pooled data from two biological replicates. Error bars in (**C**–**F**) indicate SEM of three independent replicates. Survival curves in (**G**,**H**) depict pooled data from three independent trials. Survival data of all replicates are summarized in Tables S3–S6. EV—empty vector control. ns: *p* > 0.05, ** *p* ≤ 0.01 (RNAi*strain interaction, two-way ANOVA).

Considering previously shown stress-protectant roles of glycogen in *C. elegans* [20,23,24,25], and in view of our findings that large glycogen deposits are not important for longevity of *daf-2* mutants, we decided to test the relevance of this sugar for *daf-2* survival of osmotic, heat, oxidative and UV stress. Our data confirm the heightened resistance to all the tested stressors in *daf-2* mutants in comparison to the wild-type N2 (Figure 3, Tables S3–S6). Both *gsy-1* and *pygl-1* knockdown heavily impaired the *daf-2* mutant’s resistance to 500 mM NaCl hyperosmotic stress, reducing it by 72% and 75%, respectively, while having no effect on N2 survival (Figure 3A,B; Table S3. It is possible that the effect of glycogen is partially mediated through its supply of glucose units for trehalose synthesis. We showed that the extremely high resistance to salt stress in *daf-2* mutants is largely dependent on trehalose [40]. Although we detected very modest changes in steady-state trehalose levels in *daf-2* mutants with disrupted glycogen metabolism (Figure 2B,D), it is plausible that upon harsh osmotic stress the increased demand for trehalose could be met by its additional de novo synthesis from glycogen-derived glucose 6-phosphate.

Intriguingly, we found that *pygl-1* knockdown reduced thermotolerance in *daf-2* mutants by 42% (*p*_RNAi*strain_= 0.0023; Figure 3D; Table S4), whereas *gsy-1* knockdown caused no changes in heat stress survival (Figure 3C; Table S4). We also showed a modest decrease in resistance to heat stress in the *daf-2;tps-1;tps-2* mutant that is devoid of trehalose [40], however, this effect was less outspoken than that resulting from supressed activity of glycogen phosphorylase. Our data imply that glycogen consumption is more important than glycogen synthesis for survival of heat stress in the context of reduced *daf-2* (insulin) signalling, or that very high glycogen levels are detrimental to these worms under heat shock conditions. It is yet unclear what could be an underlying mechanism for such an effect.

Both *gsy-1* and *pygl-1* knockdown caused a small decrease in resistance to tert-butyl hydroperoxide (TBHP) in *daf-2* worms (Figure 3E,F; Table S5). In contrast with our data, Gusarov et al. [24] showed a strong decrease in survival of paraquat- and diamide-induced oxidative stress in the wild-type worms subjected to *gsy-1* RNAi. These distinct outcomes could potentially be ascribed to the different age of the worms used (day 2 adults in our study versus L4 larvae in [24]), the different type of oxidative stress applied, or the lack of statistical significance could be ascribed to type II error, and more replicates are needed to confirm our results. Finally, we show that knockdown of *gsy-1* decreased survival under UV stress to a similar extent in both strains, i.e., a 17% and 21% reduction in N2 and *daf-2*, respectively (Figure 3G; Table S6). *pygl-1* RNAi caused a modest increase in survival in both strains, but this effect was not statistically significant (Figure 3H; Table S6). In this scenario, glycogen itself could potentially have a protective role against UV-C-induced oxidative damage, by a yet unknown mechanism that is independent on *daf-2*/IIS signalling.

### 3.4. Glycogen Is an Important Energy Source during Early Starvation in the daf-2(e1370) Mutant

**Figure 4:**
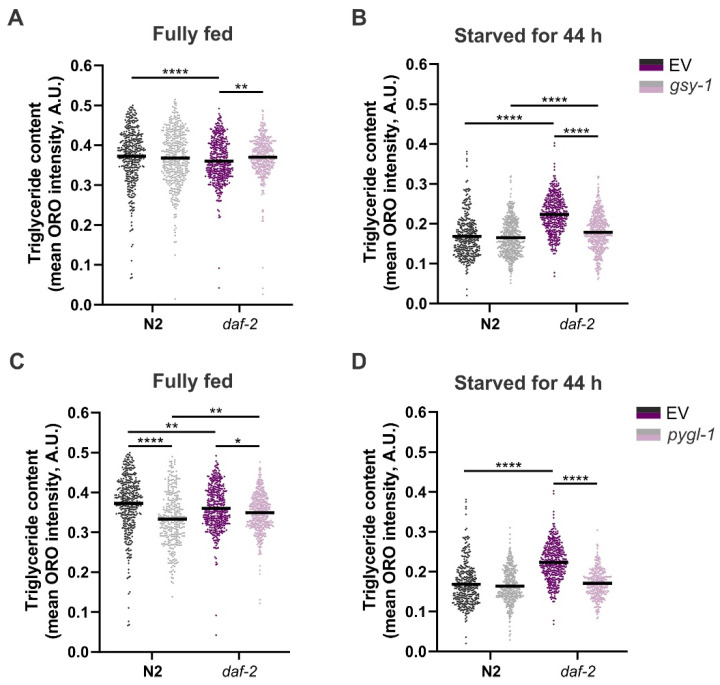
The effect of suppressing glycogen synthesis (**A**,**B**) and breakdown (**C**,**D**) on triglyceride levels in wild-type N2 and *daf-2(e1370)* mutants in fully fed condition (**A**,**C**) and after 44 h of starvation (**B**,**D**). In all graphs, three independent replicates are plotted together, with indicated mean. Each data point represents a mean ORO intensity for a single worm. EV—empty vector control. * *p* ≤ 0.05, ** *p* ≤ 0.01, **** *p* ≤ 0.0001 (Kruskal–Wallis test).

Finally, we sought to answer whether modulating glycogen metabolism would affect fat storage in *daf-2* mutants. To our surprise, *daf-2* mutants fed on EV control did not exhibit increased fat content in comparison to the age-matched N2 worms (Figure 4A,C), which is at odds with previous reports [5,57,58,59]. The exact reason for this discrepancy is currently unknown, but may be related to small differences in feeding and culturing conditions, and the age of the worms (we opted for day 2 adults based on our previous report [5]). However, a study using dark-field microscopy for fat quantification also reported comparable fat levels in N2 and *daf-2* grown at 15 °C or 20 °C [60]. To circumvent this lack of phenotype, we decided to starve the worms for 44 h. We reasoned that starvation would aid in revealing the fat phenotype in *daf-2* mutants, as we expected that in these worms, the release of energy from stored fat would be slower and more economical (dauer-like) than in the wild-type. In parallel, we evaluated the effects of *gsy-1* and *pygl-1* knockdown on fat levels in fully fed worms.

In a fully fed state, *pygl-1* RNAi caused a decrease in fat content in both strains, although more prominently in the wild-type (Figure 4C).

Mobilisation of readily available glycogen in skeletal muscles of *D. melanogaster* is a vital source of energy to support flight [61], and in *C. elegans* dauers, glycogen fuels locomotion and nictation [62], in agreement with a strong expression of *pygl-1* in the muscle tissue of worms [63,64]. The increased fat consumption in N2 worms with *pygl-1* knockdown might be attributed to a higher level of motility that would require utilization of fat as an energy source due to inability to metabolize glycogen. Conversely, *daf-2* mutants show a slow movement and largely reduced motility duration already very early in adulthood [65,66], which may lead to a milder phenotype in Figure 4C. Surprisingly, we did not observe a reduction in fat content in fully fed worms treated with *gsy-1* RNAi (Figure 4A). In the wild-type N2 this could perhaps be explained by the doubling of the amount of trehalose concomitant with limited glycogen synthesis (Figure 2B), since trehalose can also function as a glucose source to support increased energy demands [61].

While in conditions of abundant food, glycogen plays a minor role as a reservoir for energy production, it becomes vital during periods of food scarcity. *Caenorhabditis* sp. were shown to rapidly mobilize their glycogen stores during starvation [67]. Similarly, in *D. melanogaster* third-instar larvae, glycogen reserves are completely depleted after 4 h of starvation [68]. Therefore, we expected that interfering with glycogen metabolism would cause a shift toward increased fat mobilisation in starving *daf-2* worms. Indeed, in *daf-2* mutants that were exposed to *gsy-1* and *pygl-1* RNAi and subsequently starved for 44 h, fat content dropped to the levels observed in the wild-type worms (Figure 4B,D). Inhibition of *gsy-1* and *pygl-1* likely leads to the immediate burning of fat for energy during early starvation because glycogen (that would be a first carbon source to be utilized in absence of food) is either not present (or at a very low level, due to *gsy-1* inhibition) or cannot be broken down (due to *pygl-1* depletion). These data highlight the importance of large glycogen deposits in *daf-2* mutants as a source of energy during early stages of food scarcity.

### 3.5. lea-1 Is Not Important for daf-2(e1370) Longevity, but Offers Partial Protection against Osmotic, Heat and UV Stress

**Figure 5:**
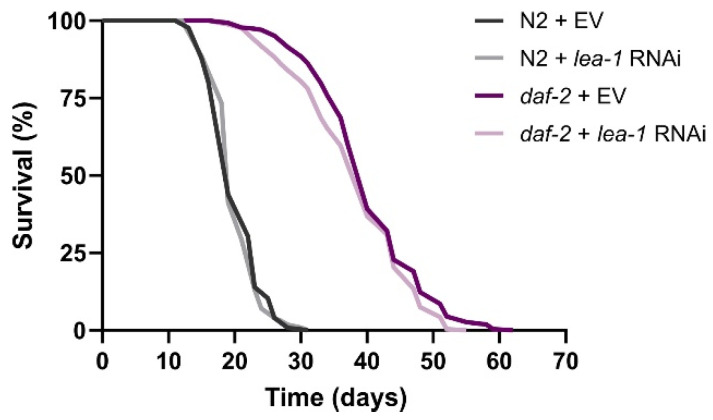
Survival curves of wild-type N2 and *daf-2(e1370)* worms subjected to *lea-1* RNAi. The graph represents pooled data from two independent experiments. EV—empty vector control. The data are summarized in Table S1.

Similar to dauers, *daf-2* mutants show elevated expression of the intrinsically disordered protein LEA-1 [5,16,39], which is a homologue of the group 3 LEA proteins found in plants. We reasoned that this is a consequence of a heterochronic activation of a dauer genetic program that is unrelated to *daf-2* longevity but could contribute to the mutant’s increased stress resistance. LEA proteins function primarily to counteract damage caused by desiccation, but they also exert protective roles against freezing, heat, and salt stress [36,37]. Their protective roles are partially attributed to the prevention of protein aggregation [69], stabilisation of cellular membranes by preventing phase transitions [70], and metal ion sequestration [36]. To test our hypothesis, we first explored the effect of *lea-1* knockdown on *daf-2* lifespan. To that end, we knocked down *lea-1* starting from hatching. In agreement with our expectations, *lea-1* was dispensable for longevity in *daf-2* mutants (Figure 5; Table S1). qPCR analysis showed that knockdown of *lea-1* by RNAi was sufficient (Figure S1).

**Figure 6:**
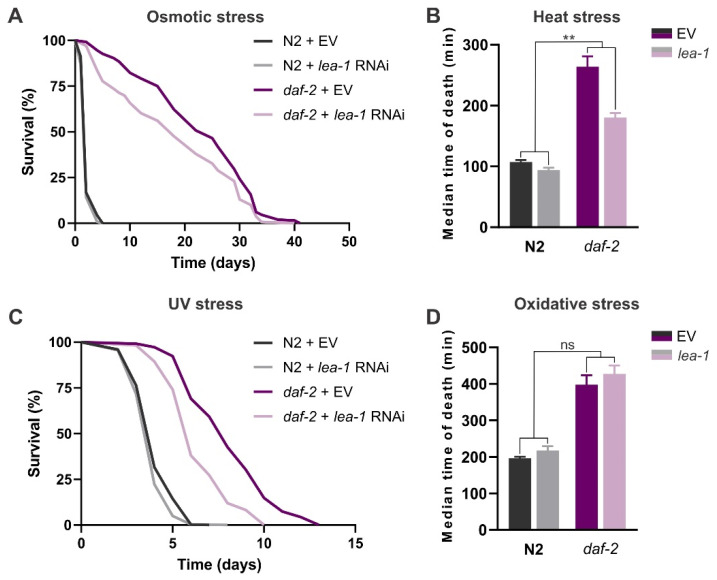
The role of lea-1 in worm stress resistance: wild-type N2 and *daf-2(e1370)* mutants with *lea-1* knockdown subjected to (**A**) osmotic stress, 500 mM NaCl; (**B**) heat stress, 40 °C; (**C**) UV stress (254 nm, 200 J/m^2^), and (**D**) oxidative stress, 0.28% tert-butyl hydroperoxide (TBHP). Survival curves in (**A**,**C**) represent pooled data from two and three independent experiments, respectively. Error bars in (**B**,**D**) indicate SEM of three independent trials. Survival data of all the stress assays are summarized in Tables S3–S6. EV—empty vector control. ns: *p* > 0.05, ** *p* ≤ 0.01 (RNAi*strain interaction, two-way ANOVA).

In their study, Erkut et al. [39] showed that *daf-2* dauers with *lea-1* RNAi knockdown were extremely sensitive to desiccation at 60% relative humidity, thus concluding that *lea-1* is essential for desiccation tolerance in these worms. These results were recently confirmed in experiments with *daf-2;lea-1* double mutants, which carry a complete deletion of the *lea-1* gene [71]. Furthermore, expression of a plant group 3 LEA protein in *E. coli* [72] and *Saccharomyces cerevisiae* [73] resulted in enhanced salt tolerance in these organisms, demonstrating effectiveness of these proteins in protection against salt stress. Consistent with these studies, we found that in *daf-2* mutants, knockdown of *lea-1* reduced survival under 500 mM NaCl by ~20% (*p*_RNAi_ = 0.0002), without affecting survival in the wild-type (Figure 6A; Table S3). Furthermore, we show that heat stress resistance in *daf-2* mutants is partially dependent on *lea-1* activity (Figure 6B; Table S4). In *daf-2*, reduction in thermotolerance due to *lea-1* knockdown is about 19% larger than that in the wild-type worms (*p* = 0.0067).

Furthermore, we found that knocking down *lea-1* partly impaired *daf-2* survival under 200 J/m^2^ UV stress (*p*_RNAi_ < 0.0001; Figure 6C; Table S6), whereas it did not affect its resistance to 0.28% TBHP (Figure 6D; Table S5). A recent study reported that expression of the hydrophilic domain of *C. elegans* LEA-1 in *E. coli* increased their resistance to oxidative stress induced by 20 mM hydrogen peroxide [74], however, we could not confirm this role of LEA-1 in our experiments. Consistent with our finding on the role of LEA-1 in UV stress response, the citrus-derived LEA protein dehydrin was shown to bind to DNA and RNA in vitro in a sequence non-specific manner [75], suggesting that these proteins might also function in the protection of nucleic acids under adverse environmental conditions. In different organisms, LEA proteins localize to multiple subcellular compartments to exert their protective roles, such as cytosol, nucleus, mitochondria, and endoplasmic reticulum (ER) [36,76]. In *C. elegans*, *lea-1* encodes 21 protein isoforms [77], and it is plausible that nuclear isoforms [78] could protect the integrity of the DNA itself during UV stress.

## 4. Discussion

In nature, *Caenorhabditis elegans* occupies humid, bacteria-rich patches of decaying plant material, e.g., rotting stems and fruits [79]. To withstand fluctuating conditions in this ephemeral habitat, *C. elegans* enters dormancy at different stages in life [79,80]. Reduction in insulin/IGF-1 (IIS) signalling due to high population density, lack of food or high ambient temperature prompts L1/early L2 larvae to develop into an alternative L3 larval stage named dauer [81]. Dauers are non-feeding, long-lived, and highly resistant to various types of stress [82,83,84]. Furthermore, they undergo a metabolic shift toward accumulation of triglycerides [85] and carbohydrates (i.e., glycogen and trehalose) [62,86], and activation of a glyoxylate shunt that allows the efficient fat-to-sugar conversion [87]. Accumulated glycogen represents an important source of energy for locomotion and nictation behaviour as dauers can actively disperse over small distances, whereas for long-distance dispersal they attach to passing invertebrate carriers [62,80]. Moreover, glycogen may be converted into trehalose, a non-reducing disaccharide that is crucial for dauer desiccation survival, by acting as a protein and membrane stabilisation agent [86]. Trehalose can also act in synergy with late embryogenesis (LEA) proteins to exert its protective function [38]. A *C. elegans* LEA homologue encoded by *lea-1* was shown to be essential for dauer desiccation tolerance [39]. Similarly, long-lived and stress-resistant IIS *daf-2(e1370)* mutants accumulate vast amounts of glycogen [5,20], and show increased protein abundance of *lea-1* [5,16].

In this study, we showed that elevated glycogen levels are not required for longevity of *daf-2* mutants. Gusarov et al. [24] reported a negligible effect of *gsy-1* and *pygl-1* on *daf-2* lifespan, i.e., *gsy-1* RNAi decreased *daf-2* lifespan by ~5%, whereas *pygl-1* RNAi caused a further extension of lifespan by ~7%. In contrast with our data and the study of Gusarov, Seo et al. [26] found that in *daf-2* mutants treated with *gsy-1* RNAi mean lifespan was further increased by ~26%. However, the latter effect appears to be independent of *daf-2(e1370)* mutation, since a similar increase in the mean lifespan due to *gsy-1* knockdown was also observed in the wild-type N2 worms [26] (N2 lifespan was also extended by *gsy-1* RNAi in [24]). Interestingly, the lifespan-extending effect of *gsy-1* suppression was attributed to a DAF-16-dependent metabolic shift toward increased storage of glucose in the form of trehalose, instead of glycogen [26]. In the wild-type N2, we detected an approximately 2-fold increase in trehalose content due to *gsy-1* knockdown, which is an even larger increase than that reported by [26]. However, the N2 lifespan was only extended by 4.7% (and this effect did not reach the 0.05 significancy level). This agrees with our earlier findings that showed no decrease in lifespan both in *daf-2* and wild-type worms that were devoid of endogenous trehalose either by a double mutation or a simultaneous knockdown of trehalose phosphate synthase genes *tps-1* and *tps-2* [40].

On the other hand, we uncovered that glycogen deposits in *daf-2* mutants have a predominant role in their protection against osmotic stress. Here, glycogen may primarily serve as reservoir of glucose for synthesis of osmolytes trehalose and glycerol. While trehalose could be produced from fat-derived acetyl-CoA via increased activity of the glyoxylate shunt, like in dauers [88], it is plausible that glycogen may be a faster and energetically less costly means of rapidly obtaining high trehalose levels. It was demonstrated that glycogen to trehalose conversion occurs in insects under different adverse environmental stimuli, such as during starvation in *D. melanogaster* [68], desiccation in the anhydrobiotic insect *Polypedilum vanderplanki* [89] or response to cold in the overwintering moth species *Cydia pomonella* [90]. Our data also support this idea. Namely, the decrease we observed in *daf-2* osmotic stress resistance due to *gsy-1* and *pygl-1* RNAi is comparable to what we previously reported in a *daf-2;tps-1;tps-2* mutant incapable of synthesising trehalose [40]. Consistently, another IIS mutant, *age-1*, further increases its trehalose levels during exposure to osmotic stress [91] and a similar increase in trehalose with concomitant decrease in glycogen was also shown in infectious juveniles of insect-killing nematode *S. feltiae* under desiccation stress [31,92]. Conversely, importance of glycogen-to-glycerol conversion for *daf-2* hyperosmotic survival may be very limited. It has been shown that the steady-state levels of this polyol under isosmotic conditions are not elevated in *daf-2* in comparison to the N2 [19]. Furthermore, exposure of *age-1* to 200 mM NaCl caused only a 2-fold increase in glycerol, whereas in N2 it rose rapidly in content by 15-fold [91]. In the wild-type worms under hyperosmotic stress, glycogen deposits are rapidly metabolized into glycerol via increased activity of glycerol-3-phosphate dehydrogenase (GPDH) genes, *gpdh-1* and *gpdh-2* [23]. However, it seems unlikely that such a mechanism of glycogen-mediated resistance to osmotic stress is at play in *daf-2* mutants, since deletions in both *gpdh* genes did not affect *daf-2* survival under 700 mM NaCl in a recent study [93]. It is noteworthy that, unlike us, Dues et al. [93] did not monitor the worm survival daily until all the worms died, but examined it only at one time point, 48 h after exposure to salt stress. Therefore, we cannot exclude the possibility that the effect of *gpdh* deletions on *daf-2* survival under osmotic stress might exist but is masked by exploring only one survival point very shortly after the exposure.

Furthermore, we found that *pygl-1*, but not *gsy-1* knockdown, caused a decrease in thermotolerance in *daf-2* mutants. The reasons behind such differences are not clear at the moment. In the yeast *Saccharomyces cerevisiae*, deletion of the glycogen phosphorylase GPH1 alters lipid metabolism, including decrease in phosphatidylcholine, likely leading to changes in phospholipid composition of the plasma membrane [94]. Given the importance of phospholipid remodelling during heat stress response [95], such a regulatory role of PYGL-1 in *daf-2* thermotolerance is a subject to speculation. We also showed that interfering with glycogen deposits did not influence resistance to oxidative stress in *daf-2* mutants to the extent shown before [24], whereas UV stress resistance was impaired to a similar extent both in *daf-2* and N2 worms treated with *gsy-1*, but not *pygl-1* RNAi. It is possible that glycogen has certain antioxidant properties that are important for worm survival of UV stress, but not for tert-butyl hydroperoxide (TBHP) resistance. The protective role of glycogen against the cellular damage induced by UV irradiation was, to our knowledge, only shown in human epidermal keratinocytes [96]. However, this was the enzymatically synthesized glycogen produced from starch, whose molecular structure slightly differs from that of natural glycogens [97]. Finally, we showed that the large amounts of glycogen that the *daf-2* mutant stores are an important source of energy during starvation. It is plausible that this is not limited only to starvation, but also other stresses, in which case glycogen could act as a rapidly mobilized source of energy to support energetically costly processes of sustaining cell integrity, such as damage repair, membrane maintenance, and preserving water and ion homeostasis [98].

In our study, we also showed that upregulation *of lea-1* is not required for *daf-2* longevity and resistance to oxidative stress, but contributes to the mutant’s enhanced survival under osmotic, heat, and UV stress. These results corroborate the previously shown stress-protective roles of LEA proteins across diverse groups, e.g., plants [99,100], rotifers [101], the anhydrobiotic nematode *Aphelenchus avenae* [102], and arthropods [35]. A study by Goyal et al. [38] reported that group 3 LEA protein from *A. avenae* could prevent protein aggregation during 43 °C heat stress. However, LEA protein does not act like a classical molecular chaperone, and it cannot prevent thermal aggregation of proteins when it acts alone, but only in synergy with the chemical chaperone trehalose [38]. We found that in the *daf-2;tps-1;tps-2* mutant that contains no endogenous trehalose, thermotolerance was reduced to a similar extent as in *daf-2* worms treated with *lea-1* RNAi [40]. Altogether, these data suggest that the chaperoning function of trehalose during heat stress might require functional *lea-1*. The antiaggregant role of the nematode LEA-1 protein was also demonstrated in vivo: aggregation of polyglutamine and polyalanine expansion proteins was effectively reduced in human cells stably expressing a group 3 LEA protein from *A. avenae* [102]. Interestingly, in addition to *lea-1*, there is another gene in *C. elegans*, named *dur-1*, which codes for a homologue of a LEA protein found in two rotifer species [39]. In a study by Erkut et al. [39], *dur-1* was essential for survival of *C. elegans* dauers both under mild and harsh desiccation. Furthermore, *dur-1* transcript levels were shown to be upregulated in *daf-2* mutants in a DAF-16-dependent fashion, whereas its expression was not detectable in wild-type N2 and *daf-2;daf-16* worms [103]. *dur-1* is strongly and almost exclusively expressed in the body wall muscle [64]. This is particularly interesting in light of a most recent finding that LEA-1 is specifically required in *C. elegans* body wall muscle to confer resistance to desiccation and osmotic stress [104], and poses a question of whether the two genes may act in cooperation.

In conclusion, we found that accumulation of glycogen and upregulation of *lea-1* are not required for *daf-2* longevity but contribute to its enhanced stress resistance. Glycogen specifically functions to increase resistance of *daf-2* mutants to osmotic stress, whereas *lea-1* has a broader stress-protectant function, thus conferring increased *daf-2* survival under osmotic, heat and UV stress. These data, together with our previous report on trehalose [40], indicate that the dauer-like metabolic shift that occurs in the *daf-2* mutant has a sole purpose of protection against stress and is unrelated to its longevity. Furthermore, our study corroborates previous findings that longevity and increased stress resistance, although positively correlated, can be uncoupled in the context of reduced *daf-2*/IIS signalling [93,105].

## Data Availability

Not applicable.

## Supplementary Materials

The following are available online at https://www.mdpi.com/article/10.3390/cells11020245/s1, Table S1: Summary of lifespan assays performed in this study (per biological replicate); Table S2: Trehalose and glycogen quantification; Table S3: Summary of osmotic stress assays (500 mM NaCl); Table S4: Summary of heat stress assays (40 °C); Table S5: Summary of oxidative stress assays (0.28% TBHP); Table S6: Summary of UV stress assays (200 J/m^2^)
