# Supplementary material for: Accumulation of Glycogen and Upregulation of LEA-1 in C. elegans daf-2(e1370) Support Stress Resistance, Not Longevity
Source: Cells. 2022 Jan 12;11(2):245. doi: 10.3390/cells11020245 (PMC8773926; doi:10.3390/cells11020245)
Supplement: Supplementary file 1 [file cells-11-00245-s001.zip › cells-1515670-supplementary.pdf]

Table S1: Summary of lifespan assays performed in this study (per biological replicate).

RNAi	Strain	Biological replicate	Mean lifespan ±SE (days)	Sample size
**EV (L4440)**	N2	1	18.59±0.37	125
		2	19.45±0.44	122
	daf-2	1	40.59±0.56	126
		2	42.14±0.84	130
lea-1	N2	1	18.28±0.35	125
		2	19.07±0.37	122
	daf-2	1	36.54±0.64	128
		2	41.29±0.85	125
**EV (L4440)**	N2	1	19.98±0.34	158
		2	20.04±0.33	149
	daf-2	1	40.27±0.74	140
		2	40.03±0.72	146
gsy-1	N2	1	21.29±0.29	150
		2	20.4±0.3	144
	daf-2	1	40.04±0.6	158
		2	39.05±0.65	143
pygl-1	N2	1	19.77±0.3	155
		2	19.56±0.29	157
	daf-2	1	41.22±0.61	177
		2	41.29±0.73	160

Table S2. Trehalose and glycogen quantification.

RNAi	Strain	Biological replicate	Trehalose (µg/mg protein)	Glycogen (µg/mg protein)
**EV (L4440)**	N2	1	90.21	5.32
		2	77.41	7.40
		3	65.90	5.90
	daf-2	1	220.09	17.69
		2	173.77	20.09
		3	198.99	20.69
gsy-1	N2	1	133.11	1.34
		2	176.56	1.28
		3	126.66	1.36
	daf-2	1	203.21	3.79
		2	187.59	3.57
		3	193.38	4.31
pygl-1	N2	1	75.04	33.62
		2	97.02	28.81
		3	42.75	26.74
	daf-2	1	109.06	44.75
		2	138.22	35.36
		3	202.83	-

Table S3. Summary of osmotic stress assays (500 mM NaCl).

RNAi	Strain	Biological replicate	Mean survival ±SE (days)	Sample size
**EV (L4440)**	N2	1	2.4±0.08	130
		2	2.18±0.09	120
	daf-2	1	22.78±0.76	130
		2	22.25±0.94	132
lea-1	N2	1	2.11±0.04	130

		2	2.21±0.1	131
	daf-2	1	17.71±0.92	135
		2	18.68±0.94	135
**EV (L4440)**	N2	1	2.75±0.12	120
		2	2.39±0.05	120
	daf-2	1	22.08±0.91	120
		2	20.37±0.75	137
gsy-1	N2	1	2.2±0.07	120
		2	2.35±0.04	123
	daf-2	1	5.61±0.22	130
		2	6.76±0.33	140
pygl-1	N2	1	2.73±0.12	120
		2	2.61±0.07	119
	daf-2	1	6.54±0.29	124
		2	4.8±0.24	136

Table S4. Summary of heat stress assays (40 °C).

RNAi	Strain	Biological replicate	Median time of death ±SE (min)
**EV (L4440)**	N2	1	113.66±4.95 ±0.09
		2	103.26±6.84
		3	104.76±13.24
	daf-2	1	286.58±29.14
		2	230.19±16.66
		3	274.61±22.76 ±0.15
lea-1	N2	1	100.48±1.41
		2	87.75±3.64
		3	94.00±5.88
	daf-2	1	195.08±10.43
		2	170.35±15.03
		3	175.79±3.49
gsy-1	N2	1	109.04±6.53
		2	92.58±3.73
		3	109.63±6.23
	daf-2	1	305.67±16.24
		2	230.74±1.38
		3	260.86±23.15
pygl-1	N2	1	115.14±5.82
		2	102.54±3.99
		3	103.72±5.33
	daf-2	1	186.64±18.95
		2	125.64±9.38
		3	143.38±16.58

Table S5. Summary of oxidative stress assays (0.28% TBHP).

RNAi	Strain	Biological replicate	Median time of death ±SE (min)
**EV (L4440)**	N2	1	202.37±4.77 ±0.09
		2	197.76±5.35
		3	188.18±10.04
	daf-2	1	390.79±15.86
		2	445.97±61.65

		3	357.34±21.46<br>±0.15
lea-1	N2	1	194.39±3.72
		2	228.01±3.46
		3	230.00±23.35
	daf-2	1	382.20±19.04
		2	458.00±20.23
		3	442.00±25.32
gsy-1	N2	1	177.97±4.18
		2	224.05±9.08
		3	181.01±8.97
	daf-2	1	312.40±9.92
		2	357.96±10.66
		3	214.07±8.02
pygl-1	N2	1	228.25±6.40
		2	216.75±5.73
		3	212.63±6.12
	daf-2	1	387.14±13.43
		2	349.10±14.70
		3	241.02±6.56

Table S6. Summary of UV stress assays (200 J/m^2).

RNAi	Strain	Biological replicate	Mean survival ±SE (days)	Sample size
**EV (L4440)**	N2	1	3.97±0.09	120
		2	4.12±0.08	124
		3	4.56±0.13	97
	daf-2	1	9.57±0.23	120
		2	7.83±0.14	124
		3	7.22±0.15	126
lea-1	N2	1	3.88±0.09	120
		2	3.97±0.07	126
		3	3.79±0.1	112
	daf-2	1	6.98±0.18	122
		2	6.38±0.12	120
		3	5.94±0.15	112
**EV (L4440)**	N2	1	4.09±0.11	121
		2	4.49±0.20	120
		3	4.56±0.13	97
	daf-2	1	8.03±0.18	117
		2	8.06±0.19	100
		3	7.22±0.15	126
gsy-1	N2	1	3.68±0.11	120
		2	3.85±0.16	120
		3	3.38±0.09	110
	daf-2	1	5.50±0.15	120
		2	7.23±0.15	126
		3	5.66±0.14	116
pygl-1	N2	1	4.89±0.14	123
		2	4.73±0.19	123
		3	4.46±0.14	118
	daf-2	1	8.56±0.22	120
		2	8.37±0.21	125
		3	8.04±0.21	104

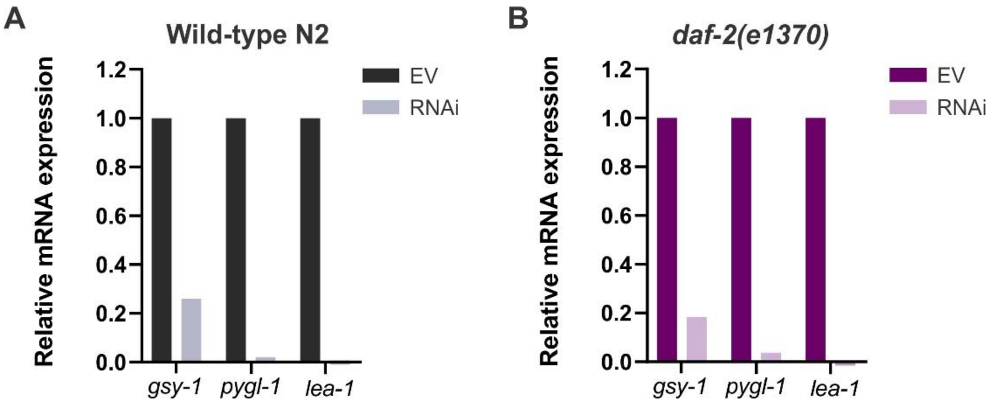

Figure S1. Quantification of *gsy-1*, *pygl-1*, and *lea-1* RNAi efficiency by RT-qPCR: in (**A**) N2 and (**B**) *daf-2(e1370)* worms. Data was normalised to the expression levels of the gene of interest in the empty vector (EV) treatment. The efficiency was assessed in a single biological replicate.
